# I Eat Healthier Than You: Differences in Healthy and Unhealthy Food Choices for Oneself and for Others

**DOI:** 10.3390/nu7064638

**Published:** 2015-06-09

**Authors:** Gudrun Sproesser, Verena Kohlbrenner, Harald Schupp, Britta Renner

**Affiliations:** 1 Psychological Assessment and Health Psychology, University of Konstanz, P.O. Box 47, Konstanz 78457, Germany; E-Mails: verena.kohlbrenner@uni-konstanz.de (V.K.); britta.renner@uni-konstanz.de (B.R.); 2 General Psychology, University of Konstanz, P.O.Box 36, Konstanz 78457, Germany; E-Mail: harald.schupp@uni-konstanz.de

**Keywords:** fake food buffet, food choice, self-other bias, optimism in eating behavior

## Abstract

The present study investigated self-other biases in actual eating behavior based on the observation of three different eating situations. To capture the complexity of real life food choices within a well-controlled setting, an ecologically valid fake food buffet with 72 different foods was employed. Sixty participants chose a healthy, a typical, and an unhealthy meal for themselves and for an average peer. We found that the typical meal for the self was more similar to the healthy than to the unhealthy meal in terms of energy content: The mean difference between the typical and healthy meals was *M*_Δ_ = 1368 kJ (327 kcal) as compared to a mean difference between the typical and unhealthy meals of *M*_Δ_ = 3075 kJ (735 kcal). Moreover, there was evidence that people apply asymmetrical standards for themselves and others: Participants chose more energy for a peer than for themselves (*M* = 4983 kJ or 1191 kcal on average for the peers’ meals *vs.* *M* = 3929 kJ or 939 kcal on average for the own meals) and more high-caloric food items for a typical meal, indicating a self-other bias. This comparatively positive self-view is in stark contrast to epidemiological data indicating overall unhealthy eating habits and demands further examination of its consequences for behavior change.

## 1. Introduction

Headlines stating that people have unhealthy eating habits such as “The way America eats is killing us” in September 2013 [1] are not surprising. Most people, experts as well as laypeople, seem to agree that eating habits in the general population are unhealthy, with too many calories and too many high-caloric foods.

However, when asking people how they gauge their own eating habits, a contrasting picture emerges. As in other life domains, many people tend to believe that they behave more positively than their average peers. For example, Sparks and colleagues [2] reported that respondents believed on average that they eat less fat and sweets in relation to others (see also [3]). In a similar vein, in a community sample, we found that only 9% believed that their own eating was less healthy than their peers’ eating, whereas 54% rated their eating as healthier than their peers’ eating [4]. This positive view of one’s own eating habits stands in contrast to epidemiological data which indicates that many people in western societies eat too much as well as too many high-caloric foods and do not eat enough fruits and vegetables. This raises the question of why we think that others eat more unhealthily than we do.

### 1.1. Self-Other Biases

The phenomenon that people think more positively of themselves than others, often to an unrealistic degree, has been demonstrated extensively (see [5,6,7,8] for reviews). This self-favoring difference in self- and others-perception has been named as “unrealistic optimism” [9], “false uniqueness” [10], or the “holier than thou” effect [11], depending on the judgment domain. Importantly, flattering self-assessments can stem from three different mechanisms. First, people might perceive themselves overly optimistically but others rather accurately (self-bias). Second, people might perceive themselves relatively accurately but others too pessimistically (peer-bias). Third, people might perceive both themselves and others inaccurately. Accordingly, to disentangle these three possible types of misjudgment, self- and others-views need to be assessed separately [5,7,8]. Whereas numerous studies have assessed self and peer related views in various judgment domains (e.g., [12]), to our knowledge, no study has yet assessed self- and peer-views separately in respect to eating. Hence, although people clearly demonstrate a self-flattering bias at the group level when they claim that they eat more healthily than their peers on average, evidence to identify whether the self-view, peer-view, or both is the source of this bias is lacking.

There are numerous psychological mechanisms underlying self-flattering biases (e.g., [5,8,13]). In particular, general or broad personality traits and behaviors typically elicit a stronger self-favoring bias than those which are more concrete and whose definition is more constrained [5]. In a similar vein, people are typically asked about hypothetical habits and behaviors, that is, how they and their peers generally behave or would behave in a given situation (see [11], see also [14]). As this represents a rather ambiguous judgment task, people might either lack the necessary information to provide accurate assessments or fail to integrate and weigh all relevant and available information [5].

Actual behavior observation reduces the ambiguity of the judgment task for participants and could, thereby, provide a more accurate picture of self-other biases than hypothetical or self-report assessment methods. Interestingly, in studies in which predicted and actual behavior was observed, people were typically more accurate in predicting others’ behaviors than in predicting their own behaviors, indicating a self-bias ([11,14], for review see [15]). For example, Epley and Dunning [11] showed that 83% of their sample predicted that they would buy a flower at a charity event, whereas they predicted that 56% of their peers would do so. After the charity event, 43% of the participants had actually bought a flower. Since the average prediction for the self was more discrepant from the observed behavior than the prediction for the peers, Epley and Dunning [11] concluded that participants showed a greater self- than peer-bias (see also [14]). However, one could argue that the observed self-bias is mainly due to poor prediction abilities for the self rather than biased perceptions of actual behavior. Recent studies suggest that people base their predictions on “ideal” behavior and, for example, attach too much weight to current intentions, whereas they fail to make realistic adjustments for situational constraints on intended actions [16].

In order to disentangle prediction errors from self-flattering perceptions of one’s own actual behavior as compared to others, we need to elicit separate behavioral probes for the self and for others. However, in current study designs this is not possible since, for a single observed behavior (e.g., likelihood of charity donation), average peer-behavior (e.g., that 43% of the sample made a donation) is an aggregate of self-behaviors and, therefore, equal to the average self-behavior. This raises the question, how can we measure self-other biases in actual behavior?

### 1.2. Measuring Self-Other Biases in Actual Eating Behaviors: Probing Behavioral Standards for the Self and an Average Peer

A first challenge in measuring self-other biases in actual behavior is to observe behaviors that are representative of what people do in their everyday lives [5,17]. Whereas natural environments have the advantage of ecological validity, a disadvantage is that food choice can be confounded by, for example, context variables such as food variety or lighting (e.g., [18]; see [19] for an overview). In contrast, laboratories offer well-controlled settings [20]. However, laboratory research faces the challenge of being representative of real-life situations; as Brunswik [21] put it, it has to have a representative design (see also [22]). Simulating the diversity of available food choices in real life in a standardized way is methodologically challenging and often conflicts with practical issues, for example, high costs, limited infrastructure, high preparation effort, and waste. To overcome these problems, an innovative method was recently developed: using replica food items [23]. With fake food items, real life, such as eating in buffet style restaurants, can be simulated under well-controlled conditions. This method has high ecological validity, has been shown to be reliable and a valid assessment of real food choices [23]. Furthermore, it allows capturing the complexity of food choice behavior that consumers face. On the one hand, how much people eat, that is the total energy content, is central for energy balance and weight maintenance (e.g., [24]). On the other hand, eating behavior comprises more than just consuming energy as an incredibly large variety of food items is available in the western world. With the fake food method, both variety and quantity can be assessed by food profiles and total energy content.

A second great challenge in measuring self-other biases in actual behavior is to separate behavior observations for the two different targets: the self and others. We would like to suggest a new method for assessing self-other biases in actual eating behavior based on the observation of three different eating situations: a typical, a healthy, and an unhealthy meal. Food choices in these three situations give information about a range of prototypical behaviors and, thus, give information which standards people apply, on average, for themselves and others in the different situations. Selected meals are a proxy for meals that are regarded as exemplary for the self and others in these situations. Hence, typical, healthy, and unhealthy behavioral standards for the self and others can be assessed.

Using a fake food buffet as standardized eating situation, it can be observed which food choices people make when they choose a typical, a healthy, or an unhealthy meal for themselves and for an average peer. These choices for the self *versus* for others enable a comparison between the average self-behavior, that is the “actual” average behavior profile (aggregated food choices for the self), and the average peer-behavior, that is the “assumed” average behavior profile (aggregated food choices for the average peer). The crucial question is whether people use different standards within and between the three different eating situations as a function of the target consumer (self *vs.* average peer). Hence, the selection of a different amount of food or other food items for the peer than for the self within one eating situation (e.g., typical meal) would indicate a self-other difference in eating standards. However, if people systematically choose more food (*i.e.*, energy), or different food (e.g., more unhealthy food items) for the peer than for the self across the three different eating situations, this would provide clear evidence of asymmetrical behavioral standards for the self and others and thereby a self-other bias in actual behavior.

### 1.3. The Present Study

The goals of the present study were twofold: (a) to investigate behavioral standards within the self and within the peer as target consumers and (b) to examine whether asymmetrical behavioral standards are employed for the self as compared to an average peer. To this end, behavioral standards for three different eating situations were assessed: a typical, a healthy, and an unhealthy meal. In addition, participants were asked to compose these three different meals for two different target consumers: for themselves and for an average peer. The present study employed a fake food buffet to simulate the diversity of available food choices in real life within a well-controlled experimental setting [23]. It was observed both how much people chose (total energy content) and what they chose (food item profiles).

## 2. Method

### 2.1. Participants

Sixty participants from the University of Konstanz took part in the study. Of these, 49 (82%) were female. Participants had a mean age of 22.4 years (SD = 5.5, ranging from 19 to 58 years) and a mean body mass index (BMI) of 22.1 kg/m^2^ (SD = 2.6, ranging from 17.9 to 30.0 kg/m^2^). Participants were recruited via flyers and received €8 for their participation.

All participants gave written informed consent prior to the collection of data. The study was conducted according to the guidelines of the Declaration of Helsinki. The procedures were performed in compliance with relevant laws and institutional guidelines. We strictly followed the German Psychological Society’s (Deutsche Gesellschaft für Psychologie) guidelines for conducting psychological studies [25] (see paragraph C.III). These are similar to those of the American Psychological Association. Moreover, the study was reviewed by the Ethics Committee (Institutional Review Board) of the University of Konstanz, Baden-Württemberg, Germany (project identification code: IRB15KN007ML; 28 May 2015). The study was judged to be exempt from full Institutional Review Board review as no threats to human health, well-being, or dignity were identified.

### 2.2. Material

**Figure 1:**
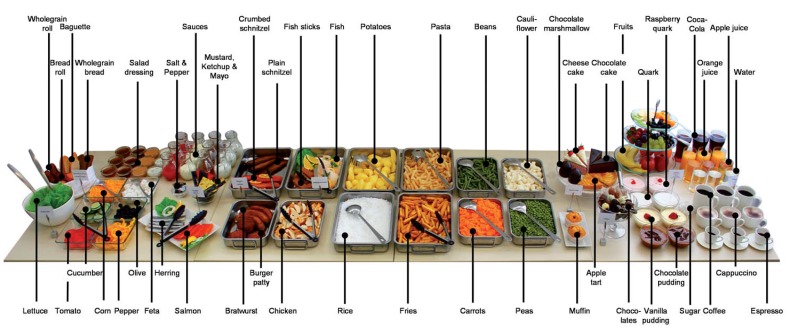
Fake food buffet containing 72 different food items.

In order to investigate participants’ food choices, a buffet with replica food items was prepared (see Figure 1). The composition of the fake food buffet aimed to represent a typical lunch buffet. An exploratory pilot study was conducted. Six participants were instructed to put together a lunch meal for themselves and for an average person of the same age and gender, that is their peer, from a fake food buffet with 55 different food items (see [26]). Participants were questioned how typical the buffet was and which items were missing or overrepresented. Overall, they found the buffet to be realistic, to include a large selection of foods, and found it easy to choose meals. However, they suggested adding sauces, dessert mousses (e.g., pudding, quark), and a larger selection of salad items. In line with their remarks, the composition of the buffet was adapted. The final fake food buffet contained a salad buffet, a selection of main courses and side dishes, a dessert buffet, and drinks. In total, it included 72 different food items (see Figure 1 and Table A1, Appendix).

For food selection, large plates (27 cm diameter), small plates (20 cm diameter), bowls (250 mL capacity), and trays (38 × 58 cm) were provided. All replica food items were manufactured by Döring GmbH, Munich, Germany. Small packets of real food condiments were also offered: Salt, pepper, mayonnaise, ketchup, and mustard. To estimate the energy content of the replica foods, the conversion factors for the weight of fake food items to corresponding real food items were calculated. Weight data was either derived from Bucher and colleagues [26] or gained by preparing corresponding real food items and taking weight measures (see Table A1, Appendix). The estimation of nutrients was based on the national database for nutritional information in Germany (Bundeslebensmittelschlüssel, [27]).

The amount of energy per meal was established by adding the caloric values of all food items selected for each meal. Additionally, to assess the quality of food choices, all food items were categorized based on a qualitative food hierarchy concerning the four food groups plant foods, animal foods, oils and fats, and drinks (German Nutrition Society, [28]; see also [29]). Within these four groups, foods were categorized as healthy, neutral, or unhealthy. The healthy food category included foods with low energy and high nutrient density as well as the recommendation to consume in high quantities. The neutral food category included foods with medium energy and medium nutrient density. The unhealthy food category included foods with high energy and low nutrient density as well as the recommendation to be consumed in small quantities. In total, 36 food items (51%) were assigned to the healthy food category, 12 food items (17%) to the neutral food category, and 22 food items (31%) to the unhealthy food category. Salt and pepper were excluded from the assignment as they were classified as spices.

### 2.3. Procedure

Participants were invited to the laboratory individually. In order to create standardized internal states of hunger, they were asked not to eat and to drink only water for two hours before participation. After giving informed consent, participants filled in a first questionnaire on demographic data. During this stage, the fake food buffet was hidden behind a movable wall to avoid potential distractions and influences.

Next, participants were instructed to compose different lunch meals from the fake food buffet. In total, participants composed six meals in two serving rounds. One serving round either consisted of successively choosing three meals for oneself (target: self) or three meals for an average peer of the same age and gender (target: peer). Within the serving rounds, participants were asked to compose a healthy meal (meal type: healthy), a meal they would typically compose (meal type: typical), and an unhealthy meal (meal type: unhealthy). Exact instructions for the typical meal type were as follows:

“Please choose a lunch meal as you would typically choose for yourself in such a buffet situation.” (target: self)

“Please choose a lunch meal as an average person of your age and gender would typically choose in such a buffet situation.” (target: peer)

For the healthy and unhealthy meal type, instructions were the same except for replacing “a lunch meal as … would typically choose in such a buffet situation” with “a healthy/unhealthy lunch meal”.

In order to control for possible carry-over and order effects, the order of choices was alternated starting either with the self- or the peer-condition. Within the self- and peer-condition, the order of meal types was alternated, too. However, the same order of meal types was applied in the two target conditions. Participants were randomly assigned to the order of choices.

After each meal was completed, the experimenter photographed the tray and weighed each food component or counted the single food items. Between the serving rounds, the buffet was refilled to initial levels, and the participant filled in a brief questionnaire on food habits. Afterwards, height and weight were measured following standardized procedures. Participants wore light indoor clothing. Height was measured without shoes to the nearest 0.1 cm using a wall-mounted stadiometer. Weight was measured using a digital scale (Omron Body Composition Monitor, BF511, Omron, Hoofddorp, The Netherland) to the nearest 0.1 kg. Finally, participants were carefully debriefed.

### 2.4. Statistical Analysis

Statistical analysis was performed using IBM SPSS statistics software, version 22.0 (IBM Corporation, Armonk, NY, USA). In order to test the research questions, ANOVAs including simple main effects and simple contrasts were computed. *p*-Values for non-orthogonal contrasts were Bonferroni-corrected. In case of violation of the assumption of sphericity, degrees of freedom were corrected using Greenhouse-Geisser estimates. All tests were based on a 0.05 significance level.

Concerning the total amount of energy, dependent variables were (1) the total amount of energy per meal and (2) the difference between the energy content of the typical meal minus the energy content of the healthy meal as well as the difference between the energy content of the unhealthy meal minus the energy content of the typical meal.

Concerning the quality of food choices, the data set was restructured, treating the 70 food items included in the healthy, neutral, and unhealthy categories as single observations and the number of participants who chose each food item within each of the six meals as continuous variable (see Figure 3). Dependent variables for these analyses were (1) the total number of participants who chose each food item and (2) the difference between the number of participants who chose each food item for the typical meal minus for the healthy meal and for the typical minus for the unhealthy meal. To ensure that the mean of each difference was positive, the difference score typical-healthy was multiplied by −1 for healthy food items and the difference score typical-unhealthy was multiplied by −1 for unhealthy food items.

Extreme values concerning the amount of energy chosen from the fake food buffet and the total number of participants who chose each food item were detected by interquartile range measures. These extreme values rendered some variables as severely skewed. After excluding extreme values, the skewness of dependent variables was acceptable (|skewness| < 1.73; |kurtosis| < 3.24; [30]). To secure our results, all analyses were repeated excluding extreme values. These analyses confirmed results reported below.

## 3. Results

### 3.1. Behavioral Standards for the Self and Others: How Much Did Participants Choose?

A 3 × 2 ANOVA with meal type (healthy *vs.* typical *vs.* unhealthy) and target (self *vs.* peer) as within-subject factors, and the total amount of energy as a dependent variable revealed significant main effects of meal type and target which were qualified by a significant meal type × target interaction (see Table 1). To follow up the interaction, additional analyses were conducted for the two factors.

**Figure 2:**
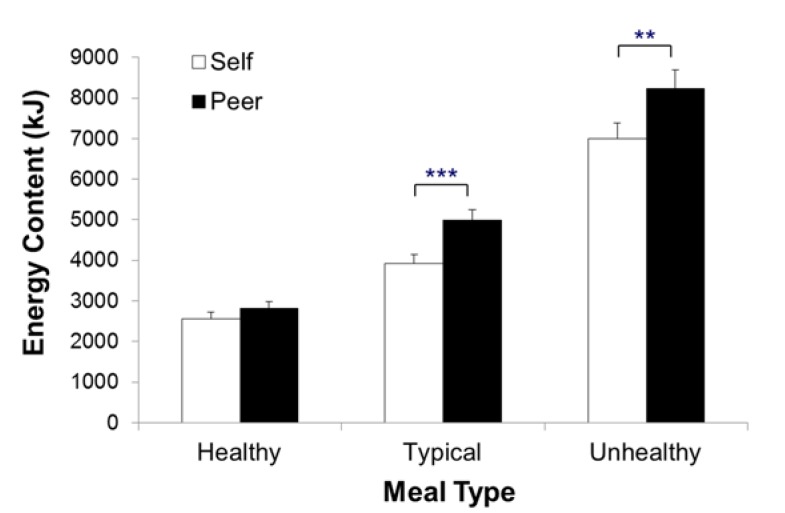
Amount of energy chosen from the fake food buffet as a function of meal type and target. Error bars indicate standard errors of the mean. *** *p* < 0.001, ** *p* < 0.01.

*Effects of meal type: Behavioral standards for the self.* All simple contrasts of meal type within the self were significant (see Table 1). As shown in Figure 2, participants chose for themselves 3929 kJ (SE = 207; 939 kcal, SE = 49.5) for a typical meal, 2561 kJ (SE = 166; 612 kcal, SE = 39.6) for the healthy meal, and 7004 kJ (SE = 373; 1674 kcal, SE = 89.2) for the unhealthy meal. Thus, participants set different behavioral standards across the three different meal types when choosing for themselves.

*Effects of meal type: Behavioral standards for the peer.* As with the meals for the self, all simple contrasts of meal type were significant for the peer (see Table 1). For an average peer, participants chose 4983 kJ (SE = 269; 1191 kcal, SE = 64.2) for a typical meal, 2824 kJ (SE = 150; 675 kcal, SE = 35.8) for a healthy meal, and 8238 kJ (SE = 460; 1969 kcal, SE = 110.0) for an unhealthy meal. Thus, participants also set different behavioral standards across the three different meal types when choosing for an average peer.

*Effects of the target person: Comparing behavioral standards for the self and peer.* Interestingly, systematic differences were seen when comparing choices between the self and peer within the three eating situations. For both the typical and unhealthy meal, participants chose significantly more energy for an average peer than for themselves (see Figure 2). However, for the healthy meal type, the amount of energy chosen was similar.

*The similarity of the typical to the healthy and unhealthy meal: A difference score analysis*. For further comparison of the behavioral standards for the self and peer, a 2 × 2 ANOVA with the meal difference score (typical-healthy *vs.* unhealthy-typical) and target (self *vs.* peer) as within-subject factors was computed (see Table 1). This revealed significant main effects of meal discrepancy and target. Specifically, within the self, the discrepancy between the typical and healthy meal was significantly smaller than the discrepancy between the typical and unhealthy meal, *M*_Δ_ = 1368 kJ; SE = 165 *vs.* *M*_Δ_ = 3075 kJ; SE = 326 (*M*_Δ_ = 327 kcal; SE = 39.5 *vs.* *M*_Δ_ = 735 kcal; SE = 78.0). Hence, participants’ typical behavioral standard was more similar to their healthy behavioral standard than to their unhealthy behavioral standard. Simple main effect analyses within the peers’ meals showed a similar pattern to the own meals: The difference between the typical and the healthy meal was significantly smaller than the difference between the typical and the unhealthy meal, *M*_Δ_ = 2155 kJ; SE = 229 *vs.* *M*_Δ_ = 3255 kJ; SE = 396 (*M*_Δ_ = 515 kcal; SE = 54.8 *vs.* *M*_Δ_ = 778 kcal; SE = 94.7). However, comparing the two targets showed a significant self-other discrepancy: When participants chose for themselves, the difference between the typical and the healthy meal was significantly smaller than when they chose for their peer. The difference between the typical and the unhealthy meal did not differ when participants chose for themselves *versus* for their peer.

**Table 1:** Results of the ANOVAS with total amount of energy chosen from the buffet as dependent variable.

Effect	*df* Effect	*df* Error	*F*	*p*	η^2^*_p_*
3 × 2 ANOVA with the factors meal type (healthy *vs.* typical *vs.* unhealthy) and target (self *vs.* peer)					
Main effect meal type	1.38	81.34	181.36	< 0.001	0.76
Main effect target	1	59	28.28	< 0.001	0.32
Meal type × target interaction	1.59	93.87	3.85	0.034	0.06
Simple contrasts within self					
Healthy *vs.* typical meal	1	59	68.63	< 0.001	0.54
Typical *vs.* unhealthy meal	1	59	88.69	< 0.001	0.60
Healthy *vs.* unhealthy meal	1	59	170.25	< 0.001	0.74
Simple contrasts within peer					
Healthy *vs.* typical meal	1	59	88.53	< 0.001	0.60
Typical *vs.* unhealthy meal	1	59	67.49	< 0.001	0.53
Healthy *vs.* unhealthy meal	1	59	183.90	< 0.001	0.76
Simple main effects target					
Healthy meal	1	59	2.35	0.131	-
Typical meal	1	59	24.65	< 0.001	0.30
Unhealthy meal	1	59	10.95	0.002	0.16
2 × 2 ANOVA with the factors meal discrepancy (typical-healthy *vs.* unhealthy-typical) and target (self *vs.* peer)					
Main effect meal discrepancy	1	59	15.87	< 0.001	0.21
Main effect target	1	59	5.66	0.021	0.09
Meal discrepancy × target interaction	1	59	1.12	0.294	-
Simple main effects meal discrepancy					
Self	1	59	19.14	< 0.001	0.25
Peer	1	59	4.64	0.035	0.07
Simple main effects target					
Typical-healthy meal	1	59	9.01	0.004	0.13
Unhealthy-typical meal	1	59	0.18	0.672	-

### 3.2. Behavioral Standards for the Self and Others: What Did Participants Choose?

A 3 × 2 × 3 ANOVA with the within-subject factors meal type (healthy *vs.* typical *vs.* unhealthy) and target (self *vs.* peer), the between-subject factor food category (healthy *vs.* neutral *vs.* unhealthy), and the number of participants who chose the food items as dependent variable revealed a significant meal type × food category interaction, target × food category interaction, and a significant three-way interaction (see Table 2). Effects of meal type and target were investigated in follow-up analyses.

**Figure 3:**
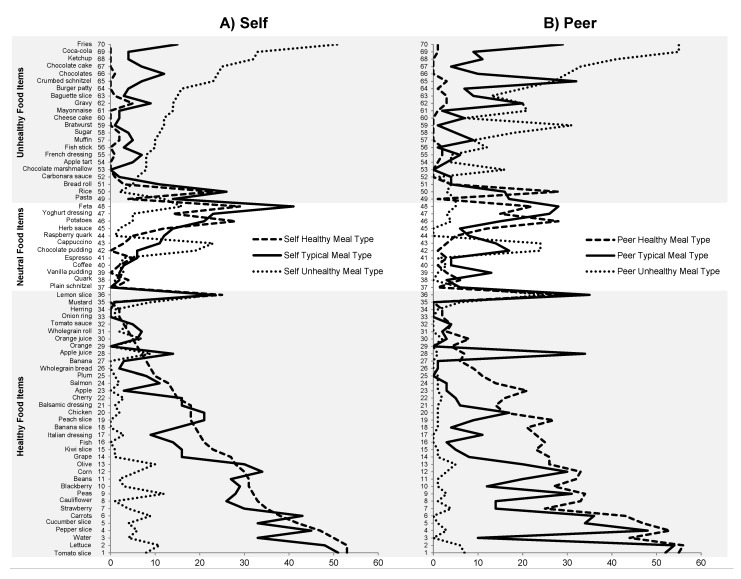
(**A**) Number of participants who chose the single food items for their own healthy, typical, and unhealthy meals (actual average behavior profile); (**B**) Number of participants who chose the single food items for the peers’ healthy, typical, and unhealthy meal (assumed average behavior profile).

*Effects of meal type: Behavioral standards for the self.* As shown in Figure 3, within the own meals, significantly more participants chose healthy food items for their healthy meal (*M* = 22; SE = 2.0) as compared to their typical meal (*M* = 19; SE = 2.0) and as compared to their unhealthy meal *(M* = 4; SE = 1.3). Moreover, significantly more participants chose healthy food items for their typical meal as compared to their unhealthy meal. For instance, 53 participants chose lettuce for their healthy meal, 48 for their typical meal, and 11 for their unhealthy meal. This pattern was reversed for the unhealthy food items: These were significantly less often chosen for participants’ healthy meal (*M* = 2; SE = 2.6) as compared to their typical meal (*M* = 7; SE = 2.6) and as compared to their unhealthy meal (*M* = 16; SE = 1.7). They were also significantly less often chosen for participants’ typical meals as compared to their unhealthy meals. In contrast, neutral food items were equally often chosen for participants’ healthy, typical, and unhealthy meals.

*Effects of meal type: Behavioral standards for the peer.* Inspecting simple contrasts revealed the same effects for the peers’ meals as for the own meals: Significantly more participants chose healthy food items for the peers’ healthy meal (*M* = 23; SE = 2.1) as compared to the peers’ typical meal (*M* = 15; SE = 2.2), and as compared to the peers’ unhealthy meal (*M* = 2; SE = 1.7). Moreover, significantly more participants chose healthy food items for the peers’ typical meal as compared to the peers’ unhealthy meal. This pattern was reversed for the unhealthy food items: These were chosen significantly less often for the peers’ healthy meal (*M* = 2; SE = 2.7) as compared to the peers’ typical meal (*M* = 9; SE = 2. 8) and as compared to the peers’ unhealthy meal (*M* = 20; SE = 2.2) as well as chosen less often for the peers’ typical meal as compared to the peers’ unhealthy meal. In contrast, neutral food items were chosen equally often for the peers’ healthy, typical, and unhealthy meals.

*Effects of the target person: Comparing behavioral standards for the self and peer.* Interestingly, comparing the meals chosen for the self *versus* for the peer revealed that even when choosing unhealthy meals, healthy food items were chosen more often for individuals’ own meals (*M* = 4, SE = 1.3) as compared to the peers’ meals (*M* = 2, SE = 1.7). Moreover, as shown in Figure 3, unhealthy food items were chosen less often for the own meal (*M* = 16, SE = 1.7) as compared to the peers’ meal (*M* = 20, SE = 2.2). For instance, only 12 participants chose Bratwurst for their own unhealthy meal, whereas 31 participants chose Bratwurst for their peers’ unhealthy meal. Moreover, when choosing typical meals, healthy food items were chosen more often for the own meal (*M* = 19, SE = 2.0) as compared to the peers’ meal (*M* = 15, SE = 2.2). This was the case, for example, for water, which 33 participants chose for their own typical meal, whereas only 10 participants chose water for their peers’ typical meals. The remaining simple main effects of target were not significant.

*The similarity of the typical to the healthy and unhealthy meals: A difference score analysis*. To further investigate these self-other discrepancies in behavioral standards, the similarity of the typical meal to the healthy and unhealthy meal was examined using difference scores. The 2 × 2 × 3 ANOVA with the within-subject factors meal discrepancy (typical-healthy *vs.* typical-unhealthy) and target (self *vs.* peer) and the between-subjects factor food category (healthy *vs.* neutral *vs.* unhealthy) yielded a significant main effect of meal discrepancy and target (see Table 2). Simple main effect analyses showed that participants’ typical meal was significantly more similar to their healthy meal (*M*_Δ_ = 3; SE = 0.8) than to their unhealthy meal (*M*_Δ_ = 15; SE = 2.1) concerning healthy food items. For instance, even more participants (*n* = 43) chose carrots for their typical meal than for their healthy meal (*n* = 38). In contrast, only nine participants chose carrots for their unhealthy meal. The simple main effect of meal discrepancy within the self was not significant for the neutral and unhealthy food category. Interestingly, simple main effect analyses for the peers’ meals revealed that the peers’ typical meals were not closer to the peers’ healthy meals than to the peers’ unhealthy meals concerning all three food categories. Even more participants (*n* = 32) chose crumbed schnitzel, for example, for the peers’ typical meal than for the peers’ unhealthy meal (*n* = 27). Moreover, the discrepancy between the typical and healthy meal was significantly smaller for the own meal (*M*_Δ_ = 3; SE = 0.8) as compared to the peers’ meals (*M*_Δ_ = 8; SE = 1.6) within the healthy food category. For instance, cauliflower was chosen 26 times for participants’ own typical meal and 33 times for their healthy meal. In contrast, cauliflower was chosen 14 times for the peers’ typical meals and 33 times for the peers’ healthy meals. Hence, the discrepancy between typical and healthy was only seven for own meals but as high as 19 for the peers’ meals. The remaining simple main effects of target were not significant.

**Table 2:** Results of the ANOVAS with the number of participants who chose the food items as dependent variables.

Effect	*df* Effect	*df* Error	*F*	*p*	η^2^*_p_*
3 × 2 × 3 ANOVA with the factors meal type (healthy *vs.* typical *vs.* unhealthy), target (self *vs.* peer), and food category (healthy *vs.* neutral *vs.* unhealthy)					
Main effects and meal type × target interaction	-	-	< 2.90	> 0.062	-
Meal type × food category interaction	2.60	86.93	30.48	< 0.001	0.48
Target × food category interaction	2	67	8.49	0.001	0.20
Three-way interaction	3.13	104.99	4.35	0.006	0.12
Simple contrasts within self					
Healthy food category					
Healthy *vs.* typical meal	1	67	13.54	< 0.001	0.17
Typical *vs.* unhealthy meal	1	67	49.02	< 0.001	0.42
Healthy *vs.* unhealthy meal	1	67	58.45	< 0.001	0.47
Neutral food category	1	67	< 5.22	> 0.075	-
Unhealthy food category					
Healthy *vs.* typical meal	1	67	21.33	< 0.001	0.24
Typical *vs.* unhealthy meal	1	67	11.71	0.003	0.15
Healthy *vs.* unhealthy meal	1	67	22.15	< 0.001	0.25
Simple contrasts within peer					
Healthy food category					
Healthy *vs.* typical meal	1	67	26.19	< 0.001	0.28
Typical *vs.* unhealthy meal	1	67	27.20	< 0.001	0.29
Healthy *vs.* unhealthy meal	1	67	57.61	< 0.001	0.46
Neutral food category	1	67	< 2.17	> 0.146	-
Unhealthy food category					
Healthy *vs.* typical meal	1	67	12.16	0.003	0.15
Typical *vs.* unhealthy meal	1	67	11.03	0.003	0.14
Healthy *vs.* unhealthy meal	1	67	24.66	< 0.001	0.27
Simple main effects target					
Healthy food category					
Healthy meal	1	67	2.75	0.102	-
Typical meal	1	67	11.01	0.001	0.14
Unhealthy meal	1	67	5.58	0.021	0.08
Neutral food category			< 0.35	> 0.555	-
Unhealthy food category					
Healthy meal	1	67	0.32	0.573	-
Typical meal	1	67	2.61	0.111	-
Unhealthy meal	1	67	11.67	0.001	0.15
2 × 2 × 3 ANOVA with the factor meal discrepancy (typical-healthy *vs.* typical-unhealthy), target (self *vs.* peer), and food category (healthy *vs.* neutral *vs.* unhealthy)					
Main effect meal discrepancy	1	67	6.42	0.014	0.09
Main effect target	1	67	7.58	0.008	0.10
Main effect food category and all interactions			< 2.21	> 0.118	-
Simple main effects meal discrepancy within self						
Healthy food category	1	67	30.04	< 0.001	0.31
Neutral and unhealthy food category	1	67	< 2.68	> 0.106	-
Simple main effects meal discrepancy within peer					
Healthy, neutral and unhealthy food category	1	67	< 2.05	> 0.157	-
Simple main effects target					
Healthy food category					
Typical-healthy meal	1	67	17.91	< 0.001	0.21
Unhealthy-typical meal	1	67	2.54	0.115	-
Neutral and unhealthy food category	1	67	< 2.23	> 0.140	-

## 4. Discussion

The present study investigated self-other biases in actual eating behavior based on the observation of behavioral standards for three different eating situations. To capture the diversity of food choices available in real life within a well-controlled setting, an ecologically valid fake food buffet was employed. The results showed that, when participants chose for themselves, a typical meal was more similar to a healthy meal than to an unhealthy meal. Hence, one’s own typical behavior was more similar to the healthy behavioral standard than to the unhealthy standard. Importantly, self-related meal choices differed systematically from choices made for an average peer, indicating asymmetrical behavioral standards for the peer and self. Specifically, participants chose less “healthy” meals for others than for themselves both regarding quantity and quality, indicating a self-other discrepancy. The results suggest that the observed self-peer discrepancy may be driven by a peer-bias which is displayed in the less favorable assumed average behavior profiles as compared to the actual average behavior profiles. In addition, the difference between the typical and the healthy behavioral standards was smaller for the self than for others. These results indicate a comparatively positive self-view which is in stark contrast to epidemiological data indicating that many people in the western world eat unhealthily.

### 4.1. Behavioral Standards for Different Eating Situations

Overall, we found different behavioral standards between the three eating situations both for the self and others. Interestingly, the typical behavioral standard for the self was relatively similar to the standard for the healthy eating situation. Some healthy food items (e.g., carrots, corn) were even chosen more often in the typical eating situation than in the healthy eating situation (see Figure 3). Our results further show relative accuracy for the healthy and unhealthy meals considering the selection of healthy and unhealthy foods. Consequently, the typical eating standard for the self was relatively healthy. This finding might appear to be in contrast to headlines and studies stating that children and adults have unhealthy eating habits [1,31,32]. However, there is evidence that overall unhealthy eating patterns result from unhealthy snacking between meals rather than main meals [33].

Another interesting result is that the similarity of the typical and healthy eating standard for the self was larger for healthy food items than for unhealthy food items. This might reflect that it is easier to consume healthy foods than to suppress consuming unhealthy foods. Specifically, Wegner’s [34] ironic process theory suggests that the suppression of a thought may ironically result in this thought becoming more prevalent. For instance, Adriaanse and colleagues [35] showed that planning not to eat unhealthy snacks ironically resulted in increased consumption.

The finding that the unhealthy and healthy eating standards were relatively accurate is in line with previous research showing that people’s perception of what is healthy, in general, agrees with official recommendations. For example, in several studies, people associated healthy eating with a high intake of fruits and vegetables [36,37,38,39]. It is important to note, however, that the number of participants who chose healthy food items for the healthy meal and unhealthy food items for the unhealthy meal varied across food items. For instance, fries and Coca-Cola were very often chosen for the unhealthy meal, whereas lettuce and tomatoes were very often chosen for the healthy meal (see Figure 3). In contrast, this effect was less clear for other food items such as rice. These variations might, in part, reflect that food items within the healthy and unhealthy category nevertheless differed in their nutritional quality and that the categorization used in the present study is only one of several existing categorizations of healthy and unhealthy foods. Moreover, stereotypes about unhealthy and healthy foods [40] might account for the clear effect for fries, Coca-Cola, lettuce, and tomatoes.

### 4.2. Behavioral Standards for the Self as Compared to for Others

We found evidence for asymmetrical behavioral standards for the self as compared to for others when it comes to a complex behavior, namely when choosing from 72 different food items. For instance, there was a self-other difference of 1054 kJ (252 kcal) for a typical meal. The found self-other bias is in line with previous findings of unrealistic optimism [9], false uniqueness [10], and the holier-than-thou effect [11]. Furthermore, our results complement and extend previous research on a self-favoring bias in self-reported healthy eating [2,3] and in the prediction of behaviors [11,14,16]. Specifically, our method allowed assessing separate behavior profiles for the self and others, which not only illustrated a self-other bias at the group level but also shed light on the sources of the self-other bias in actual behavior. Since the “assumed” average behavioral profile (aggregated food choices for the average peer) was more negative than the “actual” average behavior profile (aggregated food choices for the self), the observed self-other differences in our study can be attributed to a pessimistically biased peer-perception (peer-bias) which results in a flattering self-perception (see also [41]). As separate behavioral probes for the self and others were observed, prediction errors due to ignoring situational constraints [16] can be excluded as a contributing factor to the observed self-flattering bias. Moreover, observing actual behavior, rather than assessing self-reports of hypothetical or future behavior, rules out a lack or neglect of self-information as an underlying mechanism of the bias [5].

One potential mechanism for the observed peer-bias might be that others’ unhealthy eating behavior is more salient than others’ healthy eating behavior. When people observe others eating, these eating situations are often social. As research has shown that people eat more as well as more unhealthily [42] in social as compared to solo eating situations, people’s peer-perception might rely more often on social and thus, potentially more unhealthy eating situations. Moreover, people might engage in self-enhancement [7,43] and attribute a less favorable behavioral standard for their peers, if their own behavior is not in the desirable range. Conversely, if the own behavior is already in the desirable range (*i.e.*, the healthy eating situation), there might be no need for a behavioral social comparison standard that is less favorable than the behavioral profile of the self. This motivational mechanism is in line with our finding that, although people used different behavioral standards for the self than for others within the typical and unhealthy eating situation, they did not within the healthy eating situation.

Interestingly, despite overall asymmetrical behavioral standards for the self and others in the typical and unhealthy eating situation, “zooming” in on single food items revealed that the size of self-other differences varied across food items. For instance, large differences in behavioral standards were found for water and crumbed schnitzel within the typical eating situation. Water was chosen by 33 participants for the own typical meal but only by 10 participants for the peers’ typical meal. Similarly, there was a large difference for Bratwurst (self: *n* = 12, peer: *n* = 31) within the unhealthy eating situation. In contrast, no difference occurred, for example, for chocolate marshmallow within the typical eating situation (self: *n* = 0, peer: *n* = 0). This differentiated pattern speaks in favor of differently salient consumption stereotypes. Specifically, research in the domain of risk perception has found larger unrealistic optimism when a salient stereotype is in effect. In other words, people, on average, rated their own risk for a hazard below that of an average person when they easily pictured a clear stereotype of a typical victim of the hazard [9,44]. In line with this, a larger self-other bias in our study might display that people find it easy to imagine a person who eats these specific unhealthy foods or avoids these particular healthy foods. One conclusion from this is that public health education campaigns might facilitate instead of reducing self-other biases in eating behavior (*cf.*, [7]). Specifically, vivid presentations of eaters who avoid healthy and consume unhealthy foods may foster consumption stereotypes that are perceived as dissimilar. This might lead people to the conclusion that, although their behavior is not optimal, they eat healthily compared to others.

The observed self-other discrepancy might also result in part from the fact that our sample had a lower BMI than their average peer (*M* = 22.1 kg/m^2^ in our sample *vs.* *M* = 23.3 kg/m^2^ for an average female between 20 and 25 years [45]). Hence, our sample might consume less energy and less unhealthy foods than their average peers and further research is required to investigate self-other discrepancies in a sample which is representative in terms of body mass index (BMI).

### 4.3. Limitations

Some limitations need to be taken into account. First, our results need to be interpreted with regard to the sample of predominantly female, young adults with a normal BMI and high education from Germany. Second, the fake food buffet lacked the smell of real foods, and the quantity of leftovers could not be assessed. Third, our results provide information about the relative healthiness of meals but not about whether the meals were healthy at an absolute level. The latter requires, for example, investigation of the agreement of the meals with dietary guidelines such as MyPlate [46].

## 5. Conclusions

The present study uncovered behavioral standards for the self and others within three different eating situations by employing an ecologically valid method to capture the complexity of real life food choices. Participants’ behaviors indicate that they assume that others typically choose more energy and more unhealthy food items than they do. This “I eat more healthily than you” perception seems to be caused by a negative perception of the peer and, thus, needs to be extended to “I eat healthier than you because you are eating so unhealthily” perception. This could explain why most people agree that eating habits in the general population are unhealthy but think that they eat more healthily than others.

## Appendix

**Table A1:** Details of the food items presented on the fake food buffet.

Food Item	Real Food (g)	Fake Food	Fake Food Unit	Energy Content		Estimation Method ^a^	Food Category ^b^
				kJ/100 g	kcal/100 g		
Tomato slice	142	100	g	75	18	1	1
Lettuce	69	100	g	50	12	1	1
Water	200	1	200 mL Portion	0	0	1	1
Cucumber slice	92	100	g	50	12	1	1
Pepper slice	143	100	g	126	30	1	1
Carrots	130	100	g	138	33	1	1
Strawberry	18	1	Piece	134	32	1	1
Cauliflower	82	100	g	88	21	1	1
Peas	88	100	g	364	87	1	1
Blackberry	5	1	Piece	167	40	1	1
Beans	76	100	g	142	34	1	1
Corn	108	100	g	318	76	1	1
Olive	72	100	g	464	111	1	1
Grape	10	1	Piece	293	70	2	1
Kiwi slice	6	1	Piece	226	54	2	1
Fish	140	1	Piece	331	79	2	1
Italian dressing	44	1	50 ml Portion	1247	298	1	1
Banana slice	11	1	Piece	377	90	2	1
Peach slice	14	1	Piece	172	41	1	1
Chicken	82	100	g	481	115	1	1
Balsamic dressing	44	1	50 ml Portion	347	83	1	1
Cherry	13	1	Pair	251	60	1	1
Apple	140	1	Piece	255	61	2	1
Salmon	32	1	Slice	728	174	2	1
Plum	38	1	Piece	180	43	1	1
Wholegrain bread	31	1	Slice	828	198	2	1
Banana	126	1	Piece	377	90	2	1
Apple juice	200	1	200 ml Portion	197	47	1	1
Orange	168	1	Piece	180	43	2	1
Orange juice	200	1	200 mL Portion	180	43	1	1
Wholegrain roll	35	1	Piece	941	225	1	1
Tomato sauce	148	1	150 mL Portion	184	44	1	1
Onion ring	2	1	Piece	117	28	2	1
Herring	70	1	Slice	929	222	2	1
Mustard	10	1	10 mL Portion	360	86	1	1
Lemon slice	18	1	Piece	151	36	1	1
Plain schnitzel	125	1	Piece	849	203	2	2
Quark	250	1	250 mL Portion	305	73	1	2
Vanilla pudding	285	1	250 mL Portion	519	124	1	2
Coffee	300	1	300 mL Portion	8	2	1	2
Espresso	37	1	35 mL Portion	8	2	1	2
Chocolate pudding	285	1	250 mL Portion	397	95	1	2
Cappuccino	220	1	220 mL Portion	138	33	1	2
Raspberry quark	250	1	Piece	544	130	1	2
Herb sauce	150	1	150 mL Portion	406	97	1	2
Potatoes	113	100	g	297	71	1	2
Yoghurt dressing	50	1	50 mL Portion	607	145	1	2
Feta	110	100	g	1188	284	1	2
Pasta	87	100	g	582	139	1	3
Rice	110	100	g	527	126	1	3
Bread roll	22	1	Piece	1130	270	1	3
Carbonara sauce	150	1	150 mL Portion	1402	335	1	3
Chocolate marshmallow	23	1	Piece	1485	355	2	3
Apple tart	132	1	Piece	891	213	2	3
French dressing	50	1	50 mL Portion	1548	370	1	3
Fish stick	32	1	Piece	841	201	1	3
Muffin	37	1	Piece	1180	282	2	3
Sugar	4	1	Piece	1695	405	2	3
Bratwurst	140	1	Piece	1377	329	2	3
Cheese cake	100	1	Piece	1176	281	2	3
Mayonnaise	20	1	20 mL Portion	3109	743	1	3
Gravy	137	1	150 mL Portion	218	52	1	3
Baguette slice	24	1	Piece	1188	284	1	3
Burger patty	90	1	Piece	950	227	2	3
Crumbed schnitzel	120	1	Piece	950	227	2	3
Chocolate	10	1	Piece	1695	405	2	3
Chocolate cake	104	1	Piece	1611	385	2	3
Ketchup	20	1	20 mL Portion	460	110	1	3
Coca-Cola	200	1	200 mL Portion	197	47	1	3
Fries	82	100	g	1222	292	1	3
Salt, Pepper	-	1	Portion	-	-	-	-

Note: ^a^ Estimation method of weight data: 1 indicates *Comparison of fake food to real food*, 2 indicates *Data derived from* [26]; ^b^ Food categories were based on the German food pyramid [28]: 1 indicates *Healthy foods*, 2 indicates *Neutral foods*, 3 indicates *Unhealthy foods*.
